# The Medical Profession and National Service

**Published:** 1917-03-24

**Authors:** 


### THE MEDICAL PROFESSION AND NATIONAL SERVICE.

The pressure of the hour finds the medical profession face to face with responsibilities of high and urgent national importance. On the one hand is the repeated demand for more doctors to take service with the Army. On the other are the necessities of the civil population. To reconcile and satisfy these rival claims is an imperative duty. Moreover, in view of the limited number of practitioners available, the duty is a difficult one, and there is now practical unanimity that it cannot be discharged apart from careful and effective organisation. Failure either in one direction or the other means calamity, and the risk of this can only be avoided by an accurate adjustment of means to ends. Individual action, however well-intentioned, cannot meet this position, and therefore there must be authoritative direction and control. The object is a very direct one. It is to effect such a distribution of practitioners that both the civilian and the military needs for medical service can be met. Both patriotism and the pride of professicnal obligation urge that effort, and if need be sacrifice, shall not be wanting to enable medicine to prove itself equal to the large opportunity which confronts it at this supreme moment of national destiny.

There is no doubt at all that the medical profession is ready and anxious to meet whatever conditions are necessary for the national welfare and for the protection and care of the fighting forces. Thousands of practitioners have already put aside all personal interests for the sake of the national cause, and how well they are serving this is a matter of universal recognition. Now the depletion of the numbers of civilian practitioners has reached such a point that the demands of the Army are putting in peril the effective care of the civil population. From this position two questions emerge. The first—Is the Army using to the best advantage the doctors who have already joined its ranks? The second—Are the civilian doctors left at home fairly distributed in reference to the needs of the public? Both these questions are now in debate, and it is in our opinion a matter for congratulation that the discussion and the issues which arise out of it are in the hands of a representative medical committee. Never in these columns have we failed to recognise that great responsibilities rest upon the Army medical authorities and that a generous confidence is due to them. At the same time it is necessary to admit that in the very nature of things they regard the position from one point of view only, and it is idle to deny that their arrangements, and especially their attitude towards offers of part-time service, have justified some legitimate dissatisfaction. What must be a necessary duty of those who are now to organise the profession is to see that the whole field comes under their survey, and that the Army, equally with the civilians, effects the best possible distribution of the practitioners who are available. In agriculture and in various handicrafts such an attitude towards the War Office has been found essential to the nation's needs, and the principle is not less appropriate in relation to medicine.

With organisation comes the discussion of voluntary service or compulsion. In the minds of wise men circumstances modify opinion, and we fancy that many who in pre-war days held strongly for individual freedom are more than ready in the present emergency to vote for authoritative command. Indeed, the unanimity with which professional opinion has expressed itself in this direction is remarkable. For ourselves, we find the chief defence of compulsion in the conviction that by it alone can any approximation to equality of sacrifice be secured. And since war is no longer the clash of professional armies but the conflict of national wills every citizen has some place in the struggle. To leave the choice of this place to the individual judgment is to expect from human nature more than can reasonably be asked at its hands.

The positions here defined arise out of the war and in affirming them we admit the sacrifices and inconveniences they involve. We grant them not as good in themselves, but as justified by the emergency. When this emergency has ceased, the measures necessary to meet it will have lost their excuse. In a word, what is allowed in war must not be quoted as a precedent for the spacious days of peace.

